# Temporal evolution of the performance evaluation of the laboratories at Spanish nuclear power plants in water samples

**DOI:** 10.1007/s10661-025-13851-8

**Published:** 2025-03-21

**Authors:** José Antonio Suárez-Navarro, Virginia Peyres, Ana Isabel Sánchez-Cabezudo, Nuria Navarro, Victor Manuel Expósito-Suárez, Jordi Español, José Manuel Arteaga, Manuel Brun, Rosaura Miret, Ana Llorente, Mercedes Ibañez, J. F. Benavente

**Affiliations:** 1 Centro de Investigaciones Energéticas, Medioambientales y Tecnológicas (CIEMAT), Avenida Complutense 40, 28040 Madrid, Spain; 2 Central Nuclear de Ascó, 43791 Ascó, Spain; 3 Central Nuclear de Almaraz, 10300 Navalmoral de La Mata, Spain; 4 Central Nuclear de Garoña, 09212 Barcina del Barco, Spain; 5 Central Nuclear de Vandellós II, 43890 Hospitalet de L’Infant, Spain; 6 Central Nuclear de Cofrentes, 46625 Cofrentes, Spain; 7 Central Nuclear de Trillo, 19450 Trillo, Spain

**Keywords:** Beta emitters, Gamma emitters, PomPlot graphs, Intercomparison, Metrology

## Abstract

This study examined the performance evaluation and analysis of the laboratories of Spanish Nuclear Power Plants over the last 9 years of the 34 editions organized by CIEMAT. The participating nuclear power plants were Almaraz, Ascó I, Ascó II, Cofrentes, Santa María de Garoña, Trillo, and Vandellós II. The radionuclides analyzed were those necessary to ensure operational radiological protection and the control of liquid effluents from a nuclear power plant. The radionuclides are classified into beta emitters: ^3^H, ^14^C, and ^90^Sr, and gamma emitters: ^241^Am, ^57^Co, ^137^Cs, and ^60^Co. The results of the z score showed that the performance in determining ^3^H was 100% satisfactory in most years, except in 2010 and 2014, where percentages of 83% and 87.5% were recorded, respectively. ^90^Sr showed greater variability with a satisfaction range between 60 and 100%. Furthermore, the gamma emitters achieved satisfactory evaluations above 93% within the studied time range. The analysis of the distributions of the relative differences between the laboratory results and the reference values confirmed that the distributions were normal according to the Shapiro–Wilk test, with a percentage of outlier values below 5%. Additionally, linear regression analysis was used to evaluate the dispersion of the results relative to the reference values through the RSE parameter, being highest for ^3^H and ^14^C. The PomPlot graphs allowed for the visualization of the relationship between the obtained results and the reference values, indicating that, although high percentages of satisfactory evaluations were achieved, certain trends in the laboratory results must be considered to detect potential future deviations in the analytical methodologies.

**Supplementary Information:**

The online version contains supplementary material available at 10.1007/s10661-025-13851-8.

## Introduction

Intercomparison exercises are an essential tool to ensure the quality of analytical determinations in a laboratory (Salminen-Paatero et al., 2021). Furthermore, the UNE-EN ISO/IEC 17025 standard recommends participation in inter-comparison exercises as part of the implementation of quality control methods that ensure the validity of tests and calibrations performed (UNE, 2017). The organizer of these intercomparison exercises must meet the following requirements: be a legal entity responsible for its activities, ensure impartiality in the execution of the different phases of the exercise, and maintain the confidentiality of the information received (ISO, 2023). In this regard, the Centro de Investigaciones Energéticas, Medioambientales y Tecnológicas (CIEMAT) has organized 34 editions of the intercomparison among laboratories of Spanish Nuclear Power Plants, in which the following nuclear power plants have participated: Almaraz, Ascó I, Ascó II, Cofrentes, Santa María de Garoña, Trillo, and Vandellós II.

The selected radionuclides were those necessary to meet the specific needs of nuclear power plants from the perspective of operational radiological protection and environmental control of effluents. The selected pure beta emitters were ^3^H, ^14^C, and ^90^Sr. The ^3^H is an element generated by ternary fission reactions, producing approximately 2.2 g/year of tritium during a year of operation in a 1000-MWe plant (Nie et al., 2021). On the other hand, ^90^Sr is formed by the fission of ^235^U at a percentage of 5.77% (Shao et al., 2018). Furthermore, ^14^C is generated in small amounts during the operation of nuclear power plants as a result of the capture of neutrons by N, C, and O present in fuels, moderators, structures, or impurities (Aquilonius & Hallberg, 2005). The remaining selected radionuclides were gamma emitters: ^241^Am, ^57^Co, ^137^Cs, and ^60^Co. Both ^60^Co and ^57^Co are produced by activation reactions, while ^137^Cs is a fission product of ^235^U (Kaeriyama et al., 2014). ^241^Am is generated from the decay of ^241^Pu produced by neutron capture of ^239^Pu (Zhang et al., 2022). The matrix used has been water, as it is essential to ensure the quality of control measurements of liquid effluents from the nuclear power plant, as well as operational measurements with this type of matrix.

The performance evaluation of an intercomparison exercise is typically conducted using various statistics such as z, ζ, or E_n_ scores (ISO, 2022). These parameters utilize reference values or employ robust parameters like the median, which is less sensitive to extreme or outlier values (Xhixha et al., 2017). Moreover, the parameters used to evaluate laboratories are often accompanied by the maximum acceptable relative bias (MARB) established by the organizer, allowing for an increased range of valid values and providing a better understanding of the participants’ performance. Our working hypothesis was that the z score parameter used for evaluating the performance of the intercomparison exercise of nuclear power plants, while correct for this type of exercise, may obscure certain trends that laboratories should consider to act quickly in response to deviations in analytical methodologies. For this reason, this study employed various statistical tools to evaluate the results obtained during the last 9 years of execution of this intercomparison exercise. The specific objectives pursued to verify our hypothesis were as follows: (i) to show the historical trend of performance evaluation for the radionuclides tested using the z score parameter applying the MARB, (ii) to verify the normality of the distributions of the relative differences of the laboratory results against the reference values using kurtosis, relative bias, and the Shapiro–Wilk test, and (iii) to check for possible deviations of the results relative to the reference values through linear regression analysis and PomPlot graphs.

## Experimental

### Sample preparation

#### Calibration methods

The calibration of reference solutions of ^3^H, ^90^Sr (in secular equilibrium with ^90^Y), ^241^Am, ^57^Co, ^137^Cs, and ^60^Co in terms of activity concentration was carried out at the Ionizing Radiation Metrology Laboratory (LMRI, in Spanish) of CIEMAT. The techniques and methods employed are described below.

The reference activities of ^57^Co and ^137^Cs were determined using the 4πγ counting method, which is based on the detection of all gamma photons from a radionuclide with a 4π geometry detector, achieving efficiencies close to 100% and significantly reducing uncertainty. The total detection efficiency, ${\varepsilon }_{tot}$, is calculated as the probability of detecting at least one photon emitted per disintegration (Pommé, 2007; Thiam et al., 2015):

1 $${\varepsilon }_{tot}=1-{\prod }_{i}\left(1-{\varepsilon }_{i}\right)$$

where ${\varepsilon }_{i}$ are the detection efficiencies of the different gamma energies. The method accounts for corrections due to self-absorption and contributions from contaminating radionuclides in the source, as well as uncertainties associated with the detector geometry and material composition. The detector used was a NaI(Tl) well detector (SCIONIX) with a lead shield covered by a layer of copper. The detector was connected to an ORTEC model 460 amplifier and an ORTEC model 927 analog-to-digital converter. The samples were prepared by adding 10–30 mg of the reference solutions onto a polyethylene film in the form of a 20-mm-diameter disk, with an active surface of 3 mm. The aliquot was weighed using high-precision Mettler-Toledo microbalance, model MX5. The total efficiencies of the detector were determined using the PENELOPE and PENUC codes to simulate the decays of the two radionuclides (^57^Co and ^137^Cs) (García-Toraño et al., 2019).

The reference activities of ^60^Co, ^3^H, and ^90^Sr (in secular equilibrium with ^90^Y) were determined using liquid scintillation counting. Two calibration methods and their corresponding measurement systems were used: a TDCR counter, capable of recording the ratio between the triple and double coincidence counting rates, and a conventional liquid scintillation counter with two photomultiplier tubes. The counting efficiencies were calculated using two methods based on the free parameter model, namely the TDCR method and the CIEMAT/NIST method.

The TDCR is an absolute method in which the activity of the source can be derived from the ratio of coincidences between three and two photomultiplier tubes $\left(\frac{{N}_{T}}{{N}_{D}}\right)$ (Broda, 2003; Broda & Pochwalski, 1992; Grau Malonda & Coursey, 1988). For a large number of events, this ratio converges towards the ratio of the counting efficiencies $\left(\frac{{\varepsilon }_{T}}{{\varepsilon }_{D}}\right)$ calculated for a specific value of the free parameter. This parameter is defined as the ratio between the effective energy (corrected by ionization quenching) deposited by the particle and the photoelectrons average emitted by the photocathode (Malonda & Garcia-Toraño, 1982). In this case, the measurements were performed using a commercial TDCR counter from HIDEX, model 300SL.

On the other hand, the CIEMAT/NIST method is a semi-empirical calibration method (Coursey et al., 1986; Malonda & Garcia-Toraño, 1982), which can be implemented in any conventional scintillation system equipped with two phototubes. The procedure for establishing the efficiency of a specific radionuclide combines theoretical calculations and experimental measurements using a tracer radionuclide, with ^3^H being the most suitable. Measurements were conducted using a Quantulus 1220™ (PerkinElmer).

The counting efficiencies in both methods were theoretically calculated using free parameter models. These methods are based on the application of a physical and statistical model of the distribution and detection of scintillation photons and require detailed information about the decay scheme of the radionuclide of interest. In this case, the latest versions of the codes developed for this purpose at the LMRI were employed: EFFY9 for pure beta emitters (^3^H and ⁹⁰Sr/⁹⁰Y) and PENNUC-NUR for radionuclides with more complex decay schemes (⁶⁰Co) (García-Toraño, 2023). The sources were prepared by adding an aliquot of the reference solutions ranging from 15 to 80 mg (weighed using a Sartorius model CUBIS II precision balance) to 15 mL of the Optiphase HiSafe 3 scintillation cocktail from PerkinElmer, in low-potassium glass vials.

The activity concentration of ^241^Am was determined using an alpha spectrometer with a grid ionization chamber, model NU14B from NUMELEC, connected to a multichannel analyzer, model 920E from ORTEC EtherNIM. The samples were prepared by adding between 15 and 60 mg of solution onto a stainless steel disk-shaped planchet with a diameter of 25 mm and a thickness of 1 mm. The aliquot was weighed using a Sartorius model CUBIS II precision balance.

#### Preparation of the ^14^C solution

The ^14^C solution was obtained from the National Physical Laboratory of the UK, which provides a certified activity for such products. The final solution was prepared by the Environmental Radioactivity and Radiological Monitoring Unit of CIEMAT (URAyVR) using gravimetry with a Mettler model AT-250 balance, which is verified annually following the procedures outlined in the UNE-EN ISO/IEC 17025:2017 standard (UNE, 2017).

#### Preparation of bottles with problem solutions

The samples analyzed in the various intercomparison exercises were aqueous samples containing different radionuclides, whose reference activities were determined as described in the “Calibration methods” section. The samples provided to each participant are of three types: (i) type “A” with a ^3^H activity concentration of less than 25,000 Bq L^−1^ and the remaining radionuclides (^90^Sr, ^241^Am, ^57^Co, ^137^Cs, and ^60^Co) with an activity concentration of less than 100 Bq L^−1^, (ii) type “B” consisting of the same radionuclides as type “A” but with activity concentrations higher by 1 or 2 orders of magnitude and double for ^3^H, and (iii) type “C,” which contains ^14^C at an activity concentration on the order of 1000 Bq L^−1^. Table 1 shows the typical ranges of activity concentrations in the different samples.

**Table 1:** Activity concentrations of ^3^H, ^90^Sr, ^14^C, and gamma emitters (^241^Am, ^57^Co, ^137^Cs, and ^60^Co) in the three types of samples provided to the different participants

Radionuclide	Sample type “A”	Sample type “B”	Sample type “C”
	Activity concentration range (Bq L^−1^)	Activity concentration range (Bq L^−1^)	Activity concentration range (Bq L^−1^)
^241^Am	20–50	500–900	
^137^Cs	30–80	1000–2000	
^60^Co	50–100	3000–4000	
^57^Co	50–100	2000–3000	
^90^Sr	40–80	1500–2500	
^3^H	15,000–25000	45,000–55000	
^14^C			500–1500

Samples of type “A” and “B” were prepared in 1-L borosilicate glass bottles by the LMRI. Each bottle contained a 0.1 M HCl solution with different stable carriers added: Sr^2^⁺ (SrCl₂·6H₂O 75 mg L⁻^1^), Y^3^⁺ (YCl₃·6H₂O 75 mg L⁻^1^), Cs⁺ (CsCl 150 mg L⁻^1^), and Co^2^⁺ (CoCl₂·6H₂O 150 mg L⁻^1^). On the other hand, the type “C” sample contained a 0.1 M NaOH solution.

### Performance evaluation

The performance evaluation is conducted using the z score statistic, which is given by the following expression:

4 $$z=\frac{{x}_{lab}-{x}_{ref}}{{\sigma }_{p}\cdot {x}_{ref}}$$

where ${x}_{lab}$ is the activity concentration reported by the laboratory, ${x}_{ref}$ is the reference activity concentration of each radionuclide obtained as described in the “Sample preparation” section, and ${\sigma }_{p}$ is the standard deviation of the exercise established as maximum acceptable relative bias (MARB). The assigned MARB values in the different intercomparison exercises were 14% for ^3^H, 20% for ^14^C, and 12% for the remaining radionuclides. The value ${x}_{lab}$ is determined as the weighted mean of the results provided by the laboratories, as the participating laboratories report all the replicates they perform on the samples. Satisfactory evaluations were those that obtained $z$ score values less than 2, acceptable for values between 2 and 3, and unsatisfactory for values greater than 3. The $z$ score obtained during the period from 2010 to 2023 has allowed for the representation of the temporal evolution of the performance of the different laboratories.

The evaluation of the interlaboratory comparison exercise was carried out by assessing the photopic peaks established by the organizer (CIEMAT) of the gamma emitters added to the samples: ^241^Am (59.54 keV), ^57^Co (122.06 keV), ^137^Cs (661.66 keV), and ^60^Co (1173.23 keV and 1332.49 keV). This criterion differs from other interlaboratory comparison exercises where the organizer allows the laboratories to select the photopeaks at their discretion. This approach was adopted to evaluate the necessary parameters for determining the activity concentration more uniformly (data from the libraries with the laboratories’ nuclear data and parameters for calculating the area of the photopic peaks). Furthermore, for ^60^Co, determining two activities also allows verification of the reproducibility of the activity concentration determinations.

### Study of the distributions of the relative differences between laboratory values and the reference value

The relative differences, RB(%), were determined to study the distribution of accuracy achieved by the participating laboratories and to observe the relationship between the values themselves and the true value. The expression for RB is given by the following formula:

3 $$RB=\frac{{x}_{lab}-{x}_{ref}}{{x}_{ref}}$$

The RB(%) values obtained for the different radionuclides tested were represented using a violin plot created with the RStudio libraries (version 2024.09.0 Build 375) *dplyr*, *ggplot2*, *ggbeeswarm*, and scales. The violin plot includes the mean and median, with outlier values marked outside the interquartile range of the distribution. Outliers were identified using box-and-whisker plots. These outlier values are reflected in the violin plot with a bright green box. The distributions of the RB values were evaluated based on the following statistical parameters: (i) kurtosis, (ii) skewness, and (iii) the Shapiro–Wilk test, obtained using the moments and *stats* libraries in RStudio. The Shapiro–Wilk test determined whether the RB values followed a normal distribution. A normal distribution was indicated by a Shapiro–Wilk test value exceeding the significance level of 0.05. Distribution kurtosis were used for data categorization as platykurtic (< 3), mesocurtic (equal to 3), or leptokurtic (> 3). On the other hand, the skewness parameter was used to check whether the distribution of RB exhibited any positive or negative biases.

### Evaluation of the differences between laboratory values and the reference value

The differences between the values obtained by the laboratories and the reference values for each laboratory were studied using regression analysis. The statistical parameters employed included those specific to linear fitting: slope and R^2^, as well as the following statistical parameters: (i) root square error (RSE), (ii) mean error (ME), and (iii) relative absolute error (RAE).

4 $$RSE=\sqrt{{\sum }_{i=1}^{n}\frac{{\left({y}_{lab\left(i\right)}-\widehat{{y}_{i}}\right)}^{2}}{n-p}}$$

5 $$ME=\frac{1}{n}\cdot {\sum }_{i=1}^{n}{\left({y}_{lab(i)}-\widehat{{y}_{i}}\right)}$$

6 $$RAE=100\cdot \frac{1}{n}\cdot {\sum }_{i=1}^{n}{\frac{\left({y}_{lab(i)}-\widehat{{y}_{i}}\right)}{{y}_{lab(i)}}}$$

where ${y}_{lab\left(i\right)}$ is the value from laboratory *i* and $\widehat{{y}_{i}}$ is the predicted value for observation *i* from the linear model with which the results have been compared. The information provided by these indices was used to evaluate the results of the different participating laboratories in comparison to the reference value. The RSE (root squared error) allowed for the assessment of the robustness of the results, as squaring the differences penalizes more discordant values; thus, a high RSE value would indicate greater discrepancy in the results. The ME (mean error) helped identify biases in the results where the ME was different from 0, indicating the direction of the bias. The RAE (relative absolute error) reported the error or relative difference of the results compared to the reference value as a percentage. This parameter considers the number of elements in the compared dataset, making it suitable for comparing datasets of different sizes.

### Study of the relationship between laboratory results and reference value

The relationship between the results obtained by the laboratories and the reference values was established using PomPlot graphs (Spasova et al., 2007). PomPlot graphs allow the visualization of the results of a specific analysis based on their accuracy and precision. The horizontal axis represents the relative differences between a laboratory’s result and the reference value, expressed as $\frac{D}{MAX}$, where $D= {x}_{lab\left(i\right)} - {x}_{ref}$. The vertical axis represents the relative uncertainty, expressed as $\frac{u}{MAD}$, where $u$ is the sum of ${x}_{lab\left(i\right)}^{2}$ (uncertainty of the laboratory) and ${x}_{ref}^{2}$ (uncertainty of the reference value). Both axes are expressed as multiples of the median absolute deviation (MAD) due to its robustness, being less sensitive to outliers. The MAD is defined as the median of the absolute differences between the results of the laboratories and the reference value, as expressed in (8):

7 $$MAD=Median\left|{x}_{lab(i)}-{x}_{ref}\right|$$

where ${x}_{lab\left(i\right)}$​ is the result from laboratory *i* and ${x}_{ref}$​ is the reference value.

The accuracy and precision of a result concerning the reference value are evaluated by its position on the PomPlot graph in relation to the diagonal lines of the $\zeta$ score, which are defined on the graph as $\zeta =\left|\frac{D}{u}\right|$ = 1, 2, and 3. These lines create a pyramidal structure on the graph. The location of a specific result, along with its associated uncertainty, allows it to be evaluated in relation to the reference value using the following criteria:

- Results positioned to the right of the graph indicate values higher than the reference value (${x}_{ref}$), while those on the left are lower than ${x}_{ref}$. The deviation is measured as the distance of the results from the central vertical line.
- Results located higher on the graph suggest that the uncertainty of the value is low, indicating greater precision. Conversely, results situated lower indicate less precision, evidencing a lower reliability of the result.
- Regarding the diagonals of the $\zeta$ score, results are considered to have good precision and accuracy if they fall within $\zeta$ = ± 1. There is a risk of inconsistency if they are within $\zeta$ = ± 2 due to greater variability, and they are considered inconsistent with respect to the reference value when they are located outside $\zeta$ = ± 3.

## Results

### Temporal evolution of the different analytical determinations

**Fig. 1:**
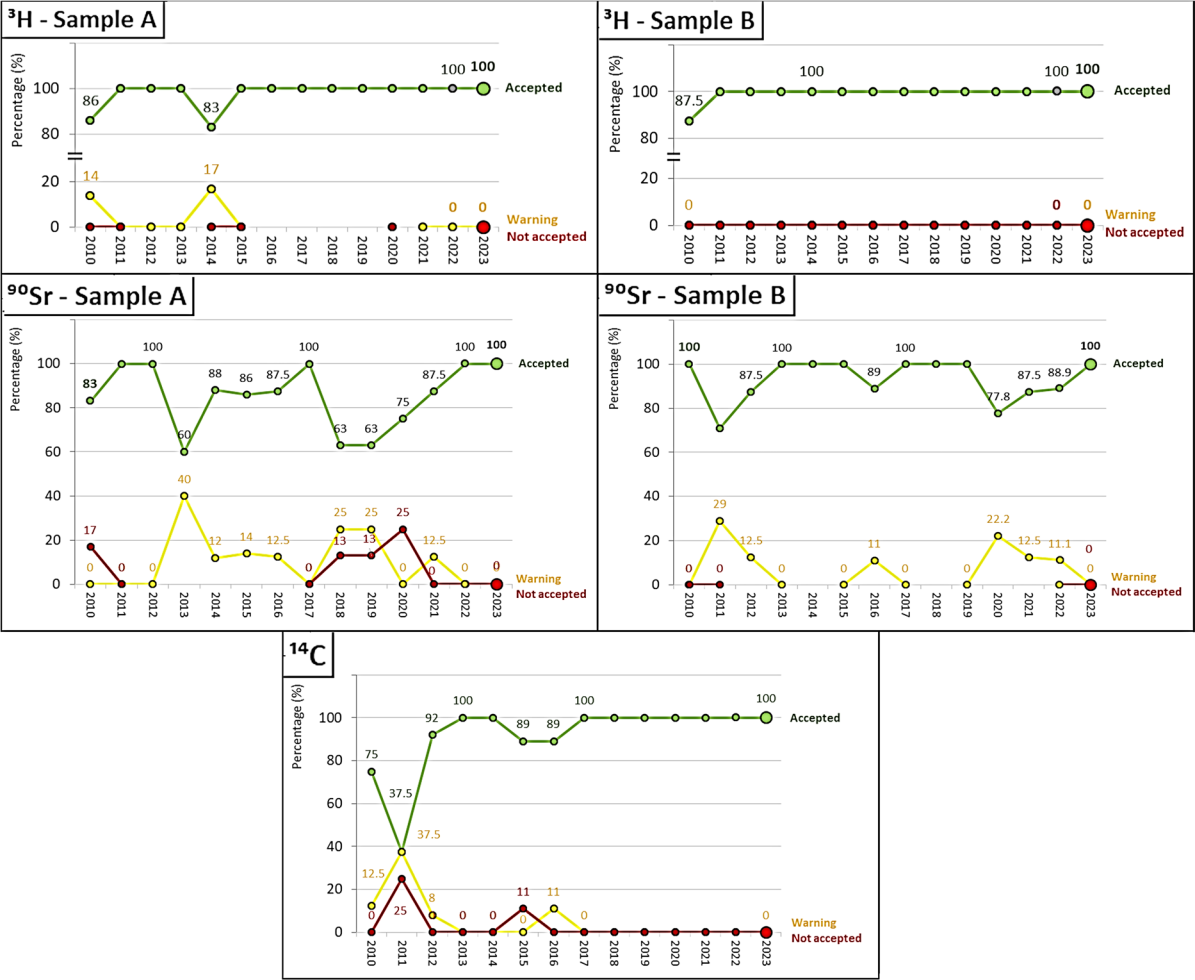
Historical performance evaluation for the analysis of pure beta emitters: ^3^H, ^90^Sr, and ^14^C for samples of type “A,” “B,” and “C.” The green lines represent Accepted scores, the yellow lines represent Warning scores, and the red lines represent Not Accepted scores

Figure 1 shows the performance evaluation for pure beta emitters (^3^H, ^90^Sr, and ^14^C) during the period from 2010 to 2023. The performance evaluation for ^3^H indicates consistently satisfactory results of 100%, except for the years 2010 and 2014 for samples of type “A” (83%) and 2010 for samples of type “B” (87.5%). The ^90^Sr demonstrates greater variability for samples of type A (ranging from 60 to 100% satisfactory values with an average of 84.3%) compared to samples of type “B” (ranging from 77.8 to 100% satisfactory values with an average of 89.6%). Furthermore, the percentage of unsatisfactory values (z score greater than 3) was also higher for samples of type “A,” reaching 25% in 2010. However, samples of type “B” did not receive any unsatisfactory evaluations, with the remaining evaluations being acceptable (z score between 2 and 3). Therefore, the results for samples of type “B,” which had a higher activity than samples of type “A,” were overall more satisfactory. Additionally, for ^14^C, it is noted that from 2017 onwards, all evaluations were satisfactory. A positive trend in satisfactory evaluations was also observed starting in 2011, which recorded the lowest percentage at 37.5%. Thus, the results for ^3^H and ^14^C were overall more satisfactory and consistent than those for ^90^Sr.

**Fig. 2:**
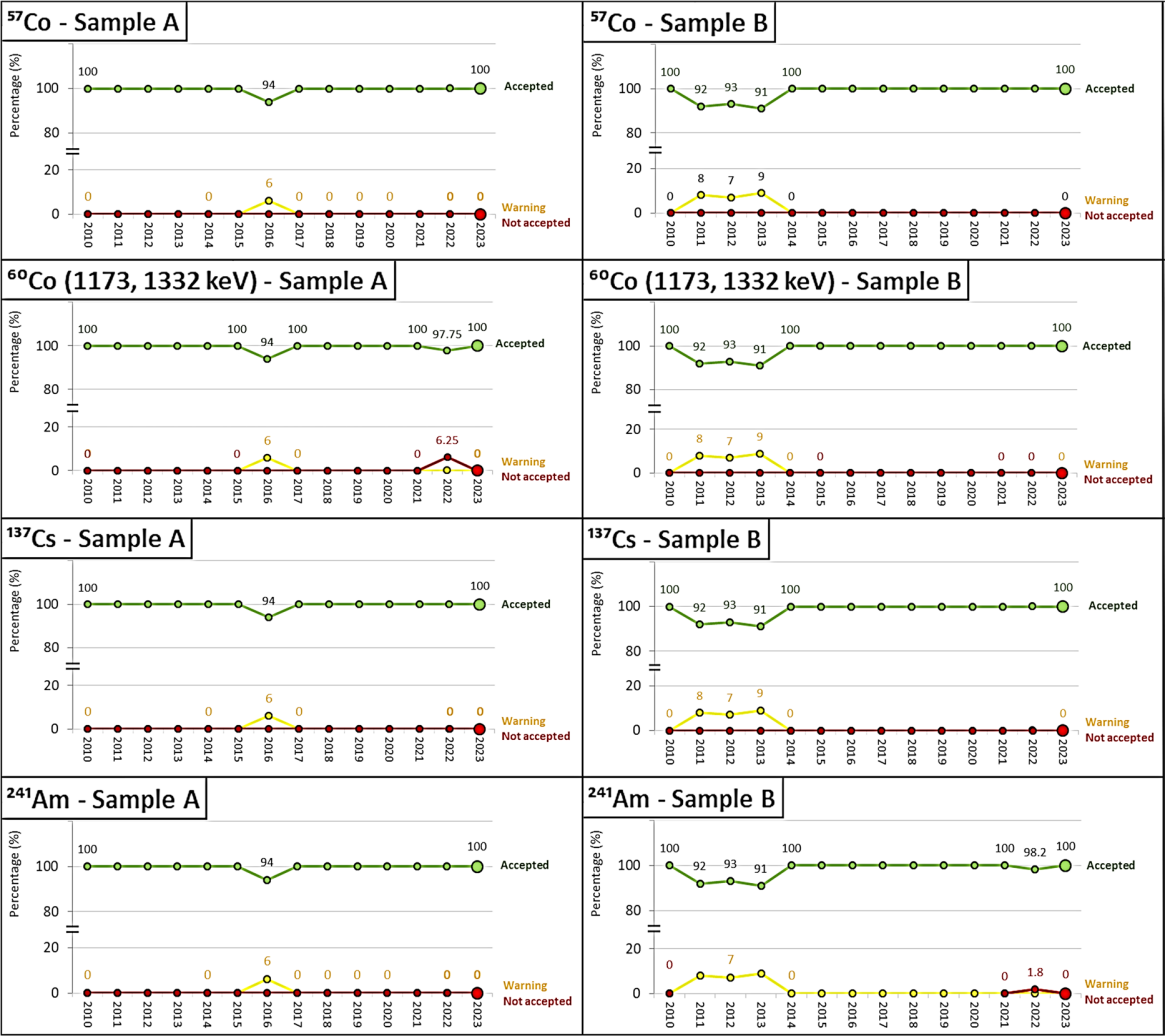
Historical performance evaluation for the analysis of beta-gamma emitters: ^57^Co, ^60^Co, ^137^Cs, and ^241^Am for samples of type “A,” “B,” and “C.” The green lines represent Accepted scores, the yellow lines represent Warning scores, and the red lines represent Not Accepted scores

Figure 2 shows the performance evolution for the gamma emitters of samples of type “A” and “B” from the different laboratories. The evaluations were equal to or greater than 91% in all years, with the rest of the evaluations being acceptable, except for the year 2022 for the ^60^Co of samples of type “A” and ^241^Am in the year 2022 for samples of type “B.”

### Study of the distributions of the relative differences between laboratory values and the reference value

**Fig. 3:**
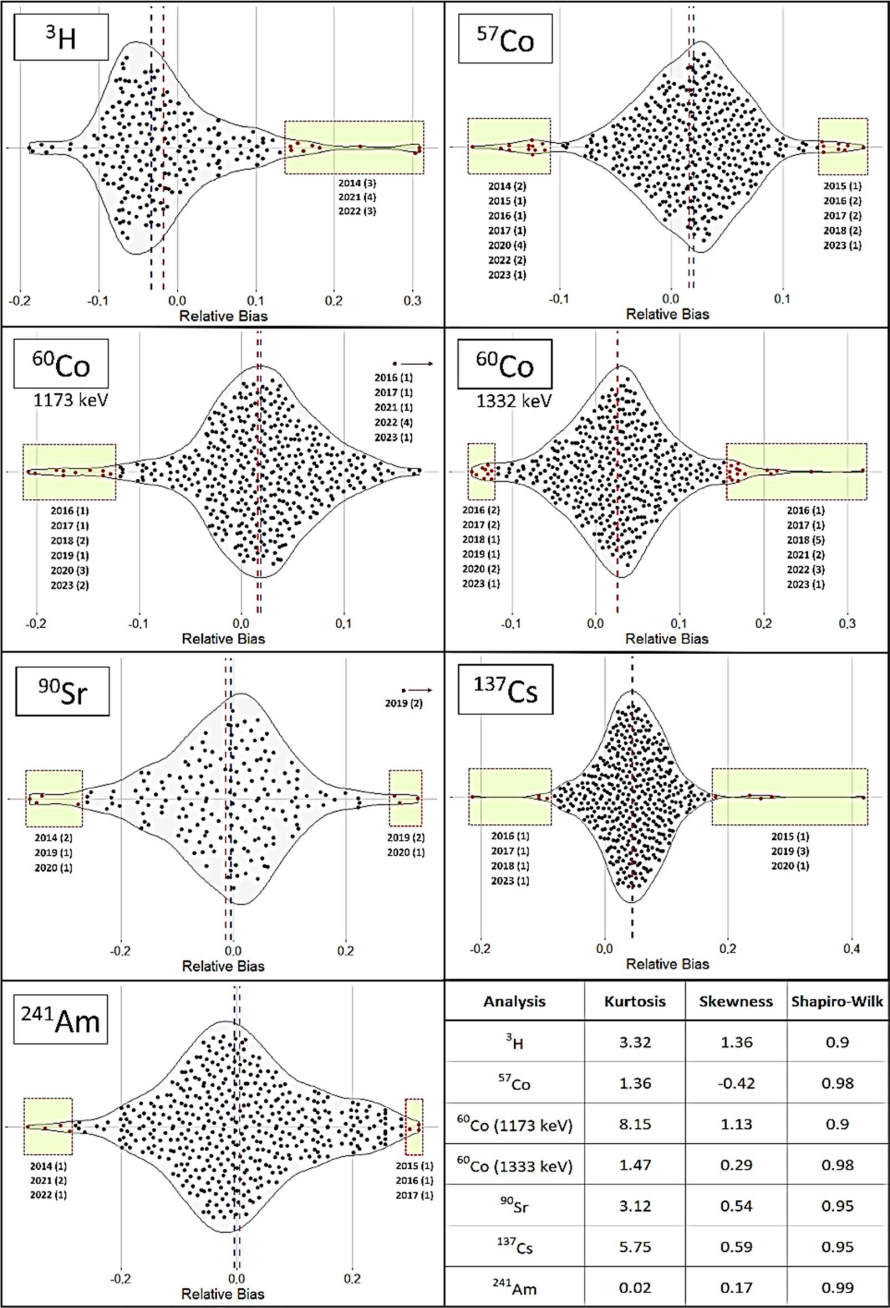
Violin plots for the relative differences of the analyses performed on samples of type “A.” The median is represented by the blue line, while the arithmetic mean is represented by the red line. The outliers are contained within the bright green boxes. Additionally, the table in the lower-right corner displays the kurtosis, skewness, and Shapiro–Wilk parameter

Figure 3 shows the violin plots with the relative differences of the results obtained by the different laboratories for samples type “A.” The blue dotted line in each plot corresponds to the median, and the red dotted line represents the mean of the relative differences. The results of the Shapiro–Wilk test indicate that all distributions were normal at a significance level of 0.05. However, the kurtosis values showed that the relative differences for ^60^Co at the photopeak of 1173 keV and ^137^Cs had a leptokurtic distribution, reflecting that all values are clustered around the mean, implying that the mean and median are practically equal. Conversely, the results for ^3^H and ^90^Sr showed mesokurtic distributions, with ^3^H having the greatest difference between the median and the mean. Furthermore, ^90^Sr had the highest relative differences, ranging from ± 0.2. Additionally, the distributions for ^57^Co, ^60^Co (1332 keV), and ^241^Am were platykurtic, indicating greater variability in the relative differences compared to the other analyses. The skewness parameter values showed significant positive skewness for ^3^H and ^60^Co (at the photopeak of 1173 keV). Outliers are detailed in Fig. 3 in the bright green boxes for each year. The box-and-whisker plots used to determine the outlier values are included in Annex 1 of the supplementary information. The analyses of ^57^Co, ^60^Co at the photopeak of 1173 keV, and ^241^Am had the most extreme outlier values as these distributions were mesokurtic. The number of outliers was 86, representing 3.33% of the relative differences. Conversely, the anomalous values were 9, representing only 0.35% of the total values. The distribution of the relative differences for ^60^Co in the photopeak of 1173 keV yielded 10 outlier values (negative) and 8 anomalous values (positive). However, the distribution of the relative differences for ^60^Co in the photopeak of 1332 keV did not yield anomalous values but recorded 22 outliers. Therefore, both photopeaks obtained an equivalent number of values outside the interquartile range of the distribution. The anomalous values obtained could explain why the kurtosis value for the photopeak of 1173 keV was 8.15, while that for the photopeak of 1332 keV was 1.47. The ^90^Sr analysis obtained 2 anomalous values, which, however, did not alter the shape of the distribution.

**Fig. 4:**
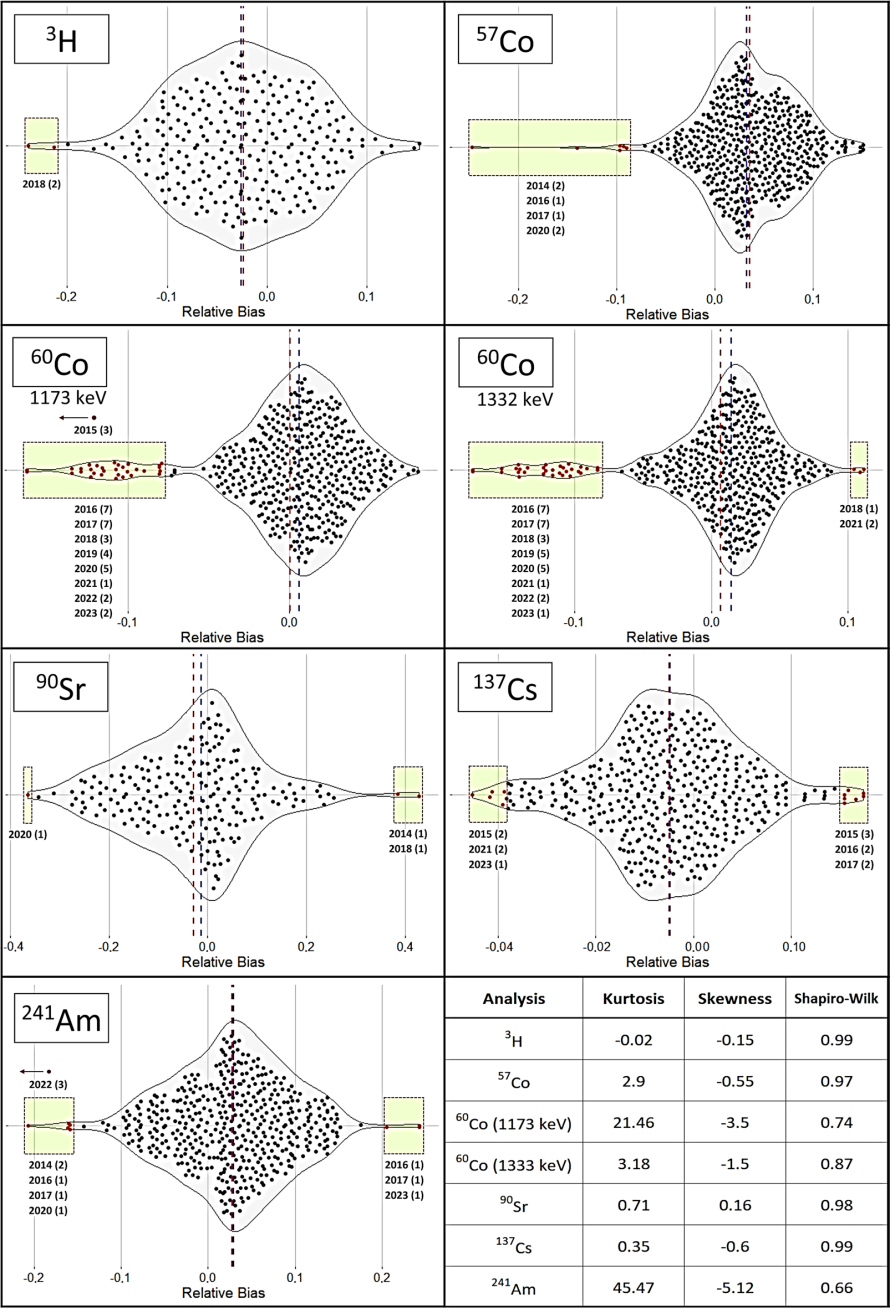
Violin plots showing the relative differences in the analyses performed on samples of type “B.” The median is represented by the blue line, while the arithmetic mean is represented by the red line. The outliers are enclosed within the bright green boxes. Additionally, the table in the lower right corner presents the kurtosis, skewness, and Shapiro–Wilk parameter

Figure 4 shows the violin plots for the relative differences obtained from the analyses performed on samples of type “B.” The distributions are normal, as indicated by the parameters of the Shapiro–Wilk test at a significance level of 0.05. The shape of the distribution was platykurtic for ^3^H, ^90^Sr, and ^137^Cs and mesokurtic for ^57^Co and ^60^Co (photopeak of 1333 keV), according to the kurtosis values. The skewness parameter showed negative biases, except for ^90^Sr, where no bias was observed. However, the differences between the mean and the median were small, confirming a homogeneous distribution of the relative differences. The number of outliers was 96, representing 3.41% of the relative differences, and the number of anomalous values was 6 (3 for ^60^Co at the photopeak of 1173 keV and 3 for ^241^Am), representing 0.21% of the total values.

**Fig. 5:**
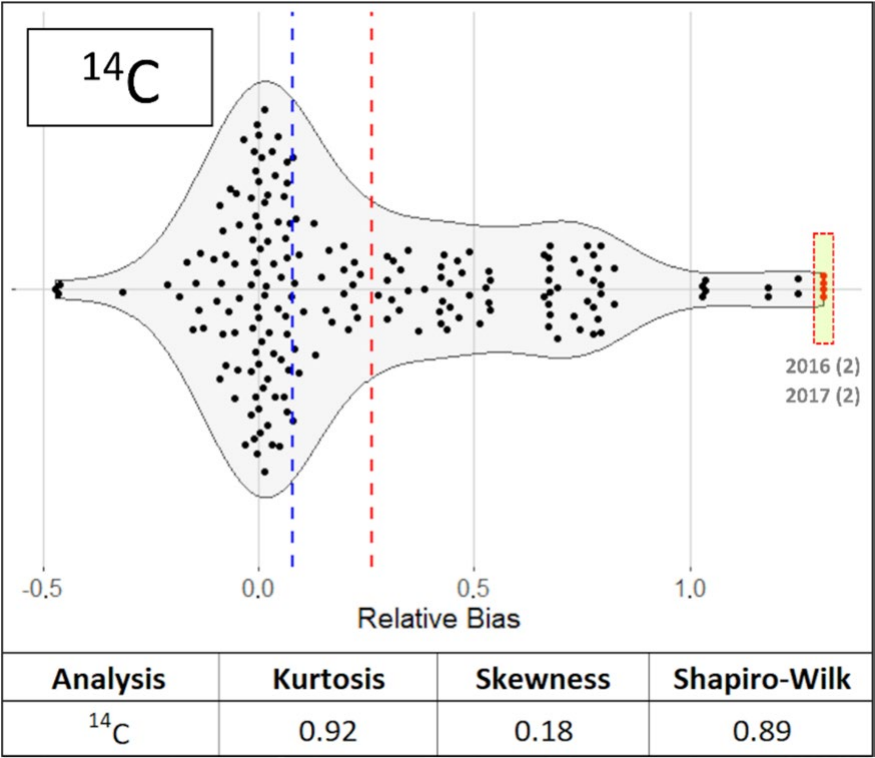
Violin plot for the relative differences obtained in the analysis of ^14^C from samples of type “C.” The blue line represents the median of the relative differences, while the red line represents the arithmetic mean

Figure 5 presents the violin plot with the relative differences for ^14^C from samples of type “C.” The Shapiro–Wilk test indicated that the distribution of the relative differences was normal. The kurtosis confirmed the platykurtic shape of the distribution. The mean and the median were different due to differences exceeding 0.2, which was equivalent to a skewness value of 0.12. The number of outliers was 4, representing 2.09% of the total values.

### Evaluation of the differences between laboratory values and the reference value

The differences obtained between the reference activity concentrations and those obtained by the laboratories were derived through linear regression analysis. Table 2 shows the values of root squared error (RSE), mean error (ME), relative absolute error (RAE%), R^2^, and the slope for the different radionuclides from samples of type “A,” “B,” and “C.” The coefficients of determination (R^2^) and the slopes of the lines reflected an agreement between the laboratory results and the reference values. However, the slope and R^2^ obtained for ^14^C indicated that the values were less concordant. This difference coincided with the RSE and RAE% values obtained for ^14^C. The RSE values for samples of type “B” were systematically higher than those for samples of type “A,” reflecting the presence of more extreme values. The ME values indicated a significant positive bias for ^3^H in samples of type “A” and a significant negative bias for ^14^C in samples of type “C.” Furthermore, the RAE% results reflected a high dispersion in the values of ^90^Sr from samples of type “A,” ^241^Am from samples of type “B,” and ^14^C in samples of type “C.”

**Table 2:** Results of the regression analysis of the different radionuclides from samples of type “A,” “B,” and “C” using the parameters root squared error (RSE), mean error (ME), relative absolute error (RAE), and R 2 and slope of the linear equation

Analysis	Sample	RSE	ME	RAE (%)	R^2^	Slope
^3^H	A	1392.0	35.0	5.4	0.994	0.9897 ± 0.0099
	B	2635.6	2.1	4.4	0.997	0.9831 ± 0.0071
⁹⁰Sr	A	28.7	−1.1	11.8	0.953	1.025 ± 0.030
	B	223.0	6.8	9.4	0.987	0.976 ± 0.015
^241^Am	A	3.0	0.2	7.2	0.993	0.988 ± 0.011
	B	89.9	2.4	19.1	0.980	0.996 ± 0.018
^13^⁷Cs	A	2.4	0.02	3.4	0.998	1.0360 ± 0.0060
	B	57.4	−1.9	2.6	0.998	1.0394 ± 0.0048
^5^⁷Co	A	3.8	0.2	4.3	0.997	0.9990 ± 0.0076
	B	99.3	0.64	3.2	0.998	1.0242 ± 0.0050
⁶⁰Co 1173 keV	A	6.2	0.1	4.5	0.994	1.0144 ± 0.0099
	B	168.8	−0.8	3.3	0.998	0.9982 ± 0.0022
⁶⁰Co 1332 keV	A	5.0	0.1	4.2	0.996	1.0197 ± 0.0080
	B	154.8	−0.1	3.1	0.998	1.0068 ± 0.0020
^14^C	C	4937.0	−23.4	28.2	0.915	1.289 ± 0.059

The uncertainties are quoted for a coverage factor *k* = 2.

### Evaluation of the accuracy and uncertainty of laboratories in relation to the reference value using PomPlot graphs

**Fig. 6:**
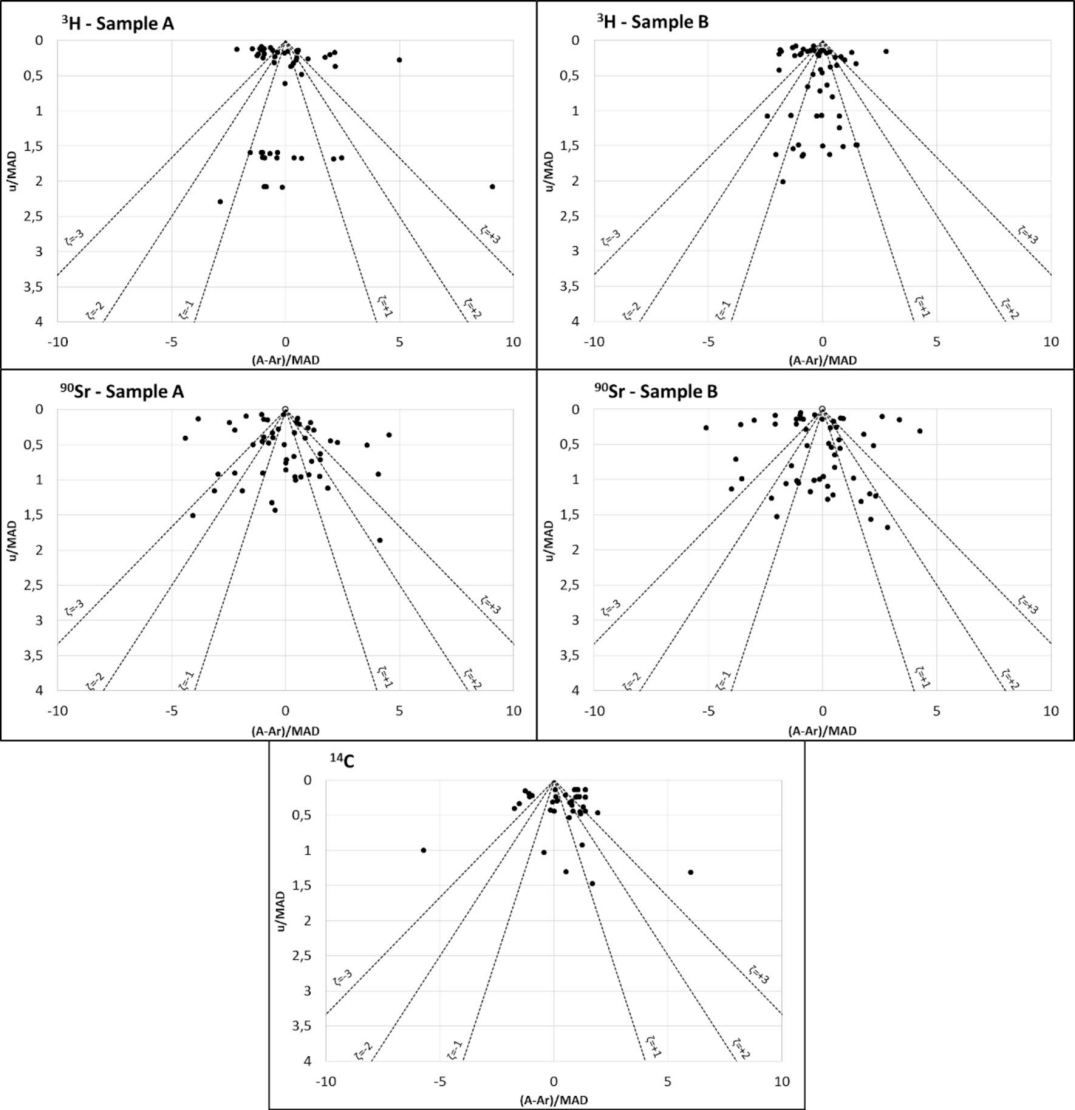
PomPlot graphs for the activity concentration of beta emitters: ^3^H, ^90^Sr, and.^14^C in samples of type “A,” “B,” and “C”

**Fig. 7:**
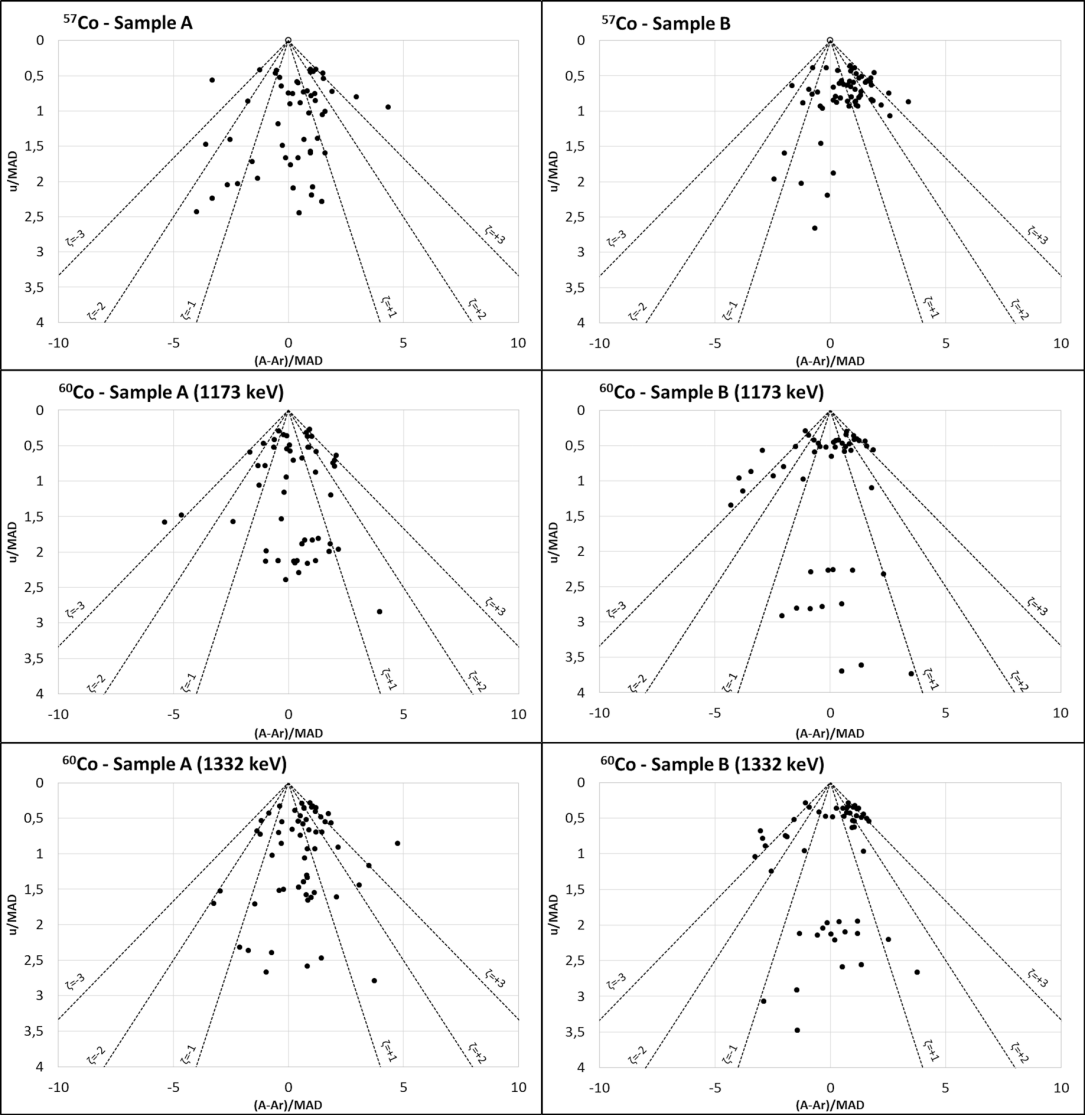
PomPlot graphs for the activity concentrations of gamma emitters with two photons at different energies in samples of type “A” and “B”: ^57^Co and ^60^Co at the photopeaks of 1173 keV and 1333 keV

**Fig. 8:**
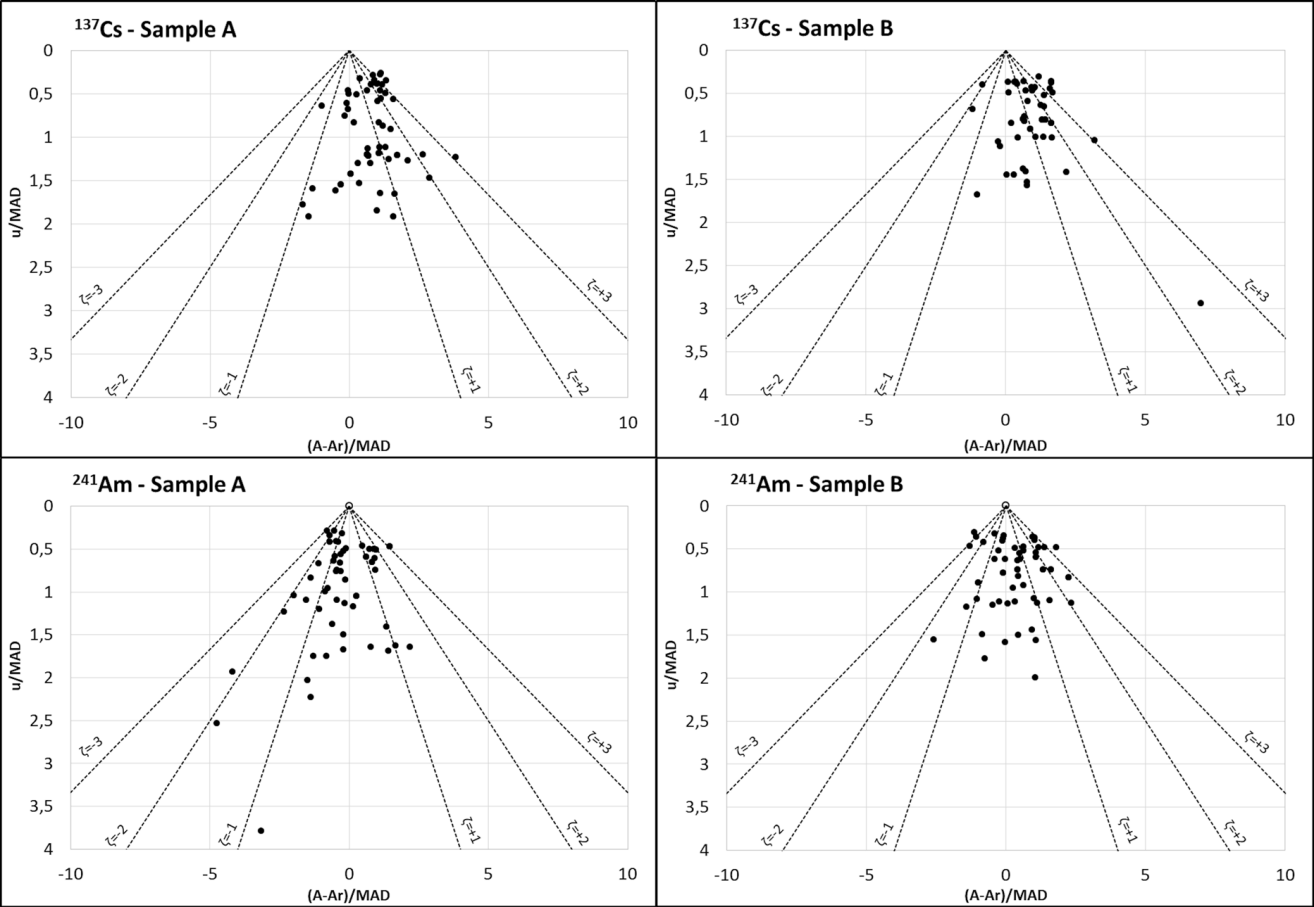
PomPlot graphs for the activity concentrations of monoenergetic gamma emitters in samples of type “A” and “B”: ^137^Cs and ^241^Am

**Fig. 9:**
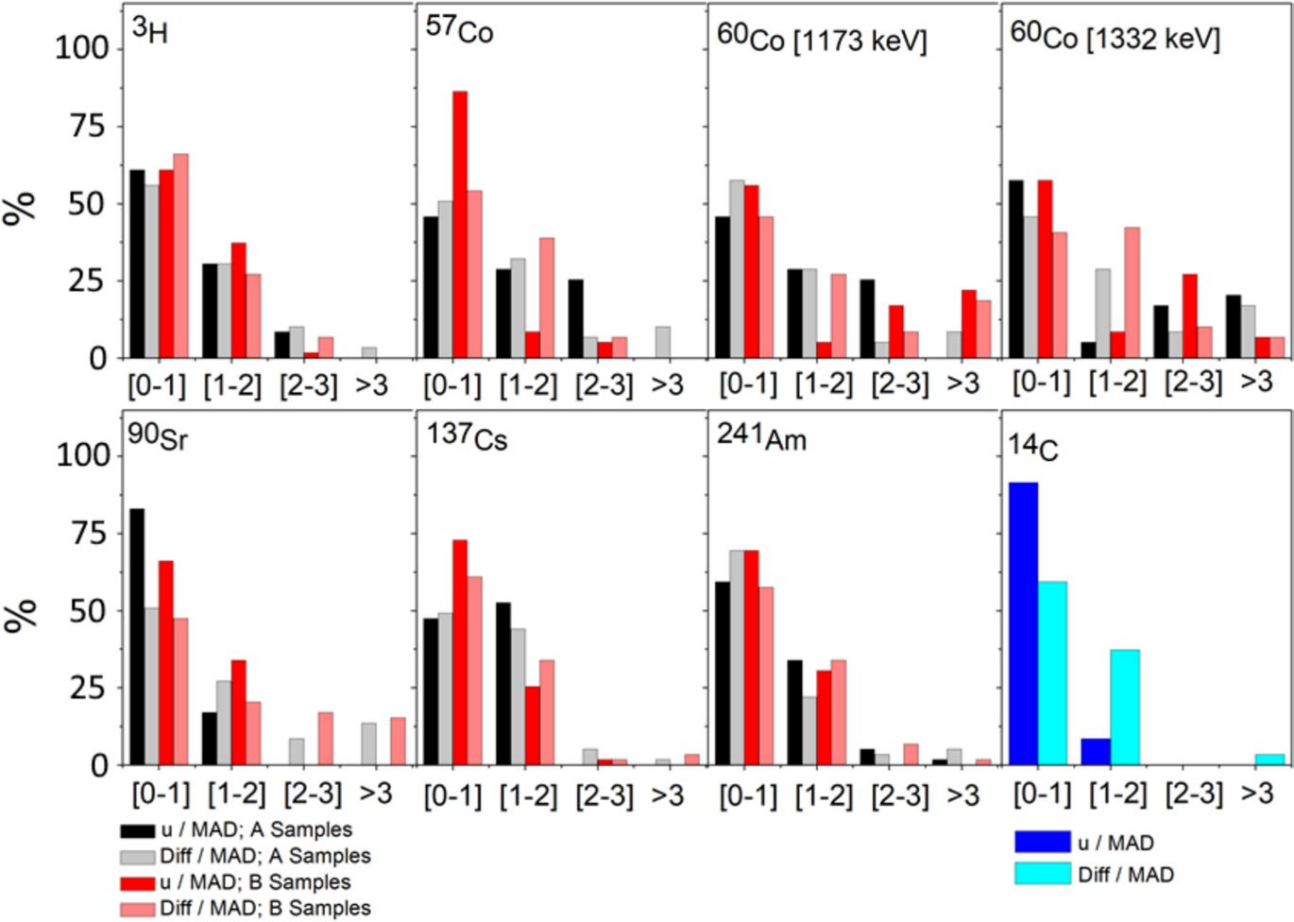
Percentages of accuracy and uncertainty obtained in the PomPlot graph for the different radionuclides in samples of type “A,” “B,” and “C” according to their position within the pyramid of ζ ≤ 1, 1 < ζ < 2, 2 < ζ < 3, and ζ ≥ 3. The color of the bars indicates: black for the accuracy of samples of type “A,” gray for the uncertainty of samples of type “A,” red for the accuracy of samples of type “B,” salmon for the uncertainty of samples of type “B,” blue for the accuracy of samples of type “C,” and light blue for the uncertainty of samples of type “C”

Figures 6, 7, and 8 display the PomPlot graphs for the beta-emitting radionuclides: ^3^H, ^90^Sr, and ^14^C; gamma emitters with gamma photons at two energy levels: ^57^Co and ^60^Co; and the monoenergetic gamma emitters (^137^Cs and ^241^Am). Additionally, Fig. 9 presents the percentages of accuracy and uncertainty obtained in the PomPlot graph for the different radionuclides in samples “A,” “B,” and “C” according to their position within the pyramid of ζ < 1, 1 < ζ < 2, 2 < ζ < 3, and ζ > 3. The PomPlot graphs indicate that no results were obtained for ζ > 3 for the three beta emitters. The ^3^H and ^14^C showed 3.39% of values exceeding 3 in samples of type “B.” Furthermore, ^90^Sr exhibited ζ > 3 values for 13.6% in sample “A” and 15.2% in sample “B.” On the other hand, the uncertainties of the monoenergetic gamma emitters (^137^Cs and ^241^Am) recorded ζ < 3, although the differences from the actual value showed significantly different values from the reference value, ranging between 1.69 and 3.39%, which can be considered low. Finally, for gamma emitters with more than one energy (^57^Co and ^60^Co), the precision scores obtained were ζ < 3 for the photopeaks of 1173 keV and 1332 keV of ^60^Co for sample type “B” (22.03% and 6.78%, respectively). Similarly, the accuracy scores with ζ > 3 were the highest compared to the other radionuclides, with a range between 6.78 and 18.64%, except for ^57^Co in sample type “B.”

## Discussion

The results obtained in this study confirm our hypothesis that the parameters used to evaluate the performance of this intercomparison exercise, while reflecting a high percentage of satisfactory assessments of the results, may conceal certain trends that laboratories must consider to act swiftly in response to deviations in analytical methodologies.

The performance evaluation conducted using the z score (expression 4) indicated that the analyses with the lowest percentage of satisfactory results were ^90^Sr in both samples of type “A” and “B,” along with ^14^C in samples of type “C.” Both ^90^Sr and ^14^C are complex radionuclides to analyze, along with ^3^H. The analysis of ^90^Sr requires complex radiochemical separations (Vajda & Kim, 2010). On the other hand, ^14^C is dissolved in 0.1 M NaOH, which causes the quenching correction to reduce counting efficiency (Hou, 2018). The remaining determinations achieved satisfactory evaluations above 90%. However, it is important to note that the value of the denominator in the z score expression is a percentage of the reference value determined from the MARB. Therefore, the range of satisfactory results could be considered a fixed value independently of the results from different laboratories concerning the reference value. For this reason, it was decided to use other types of criteria to study the results from the laboratories.

The distributions of relative differences (RB) followed normal distributions for all radionuclides analyzed by the laboratories. This condition is essential for conducting subsequent analyses correctly. The kurtosis of the distributions is a parameter that provides information on which determinations exhibit high dispersion. Determinations with a kurtosis value greater than 3 reflect greater dispersion. However, kurtosis is affected by extreme outliers, which can also result in skewness values different from 0, indicating positive or negative biases in the data. When comparing the kurtosis and skewness values of RB and their presentation in box and violin plots, some discrepancies were observed. For example, the kurtosis values for ^137^Cs and ^90^Sr in samples of type “A” were 5.75 and 3.12, indicating that ^90^Sr would have a mesokurtic distribution while ^137^Cs would be platykurtic, showing greater dispersion of values. However, when examining the RB in both cases, they were within ± 0.2, with ^137^Cs exhibiting more outliers with both positive and negative biases. Therefore, while the information provided by the RB distributions is useful for detecting biases and the shape of the distribution (Anagnostakis et al., 2004), it is challenging to quantify with standard statistical parameters. Nonetheless, the distribution information provided by the Shapiro–Wilk test and the shapes of the distributions are important for confirming the normality of the RB values, which is necessary for the application of any additional statistical tests.

Linear regression analysis was used to compare the laboratory results against the reference value (Miller & Miller, 2005). The equation used was of the form *y* = *a·x*, as the variation in reference activities was not sufficiently broad to use the y-intercept. Additionally, the parameters RSE, ME, and RAE required this adjustment to present the appropriate information. The RSE was the parameter that provided the most information regarding the dispersion of laboratory results, as it was influenced by extreme values (Expósito-Suárez et al., 2024). The highest RSE values were observed for the following: ^3^H in samples of type “A” and “B” (1392.0 and 2635.6, respectively), ^90^Sr in sample “B” (223.0), ^60^Co at the peaks of 1173 keV and 1332 keV in sample “B” (168.8 and 154.8, respectively), and ^14^C in samples of type “C” (4937.0), which exhibited the most extreme values as shown in the respective violin plots. However, the ME and RAE(%) values did not provide precise information, as they represented mean values that were offset by extreme positive and negative values, making them only useful for homogeneous distributions.

The PomPlot graphs more accurately depicted the relationship between the laboratory results and the reference activity. The graphs indicated that the uncertainty provided by the laboratories was consistent with that of the reference values (u/MAD) (Fig. 9). Only ^60^Co at the photopeaks of 1173 keV and 1332 keV for samples of type “B” recorded values of 22.0% and 6.8% for those with a ζ ≥ 3, which are considered significantly different. The observed difference was due to the fact that the result from one of the participating laboratories in 2015 was 70% between the 1173 and 1332 keV photopeaks in the “B” type sample, which was reflected in the dispersion obtained. However, the maximum difference between the results of the 1173 keV and 1332 keV photopeaks in the “B” type sample, excluding that result, was 8.2%, with an average value of 1.4%, which indicates that the results are equivalent. The (A-A_r_)/MAD values exceeded 67.8% for the ζ < 2 range. The graph demonstrated that the most accurate results, with a percentage below 10% in the ζ > 2 range, were for ^3^H and ^57^Co in samples of type “B,” both at 6.7%, ^137^Cs in samples of type “A” and “B” (6.8% and 5.1%, respectively), ^241^Am in samples of type “A” and “B” (6.8% and 8.5%), and ^14^C in samples of type “C” at 3.4%. These results were equivalent to the violin plots of the RB. This analysis focused on the individual parameters u/MAD and (A-A_r_)/MAD. The results where the uncertainty assigned to the results was less than that of the reference value, as in the case of ^3^H and ^90^Sr, obtained a less satisfactory score in the graph, placing these values outside the satisfactory value pyramid. Therefore, this graph, in conjunction with the study of the residuals, would serve as tools to provide a more accurate internal assessment of the laboratory regarding the final outcome of the intercomparison exercise.

## Conclusions

The satisfactory evaluation of an intercomparison exercise by the organizer is based on robust parameters such as the median, z, ζ, or E_n_ scores. These parameters allow for an assessment of whether the laboratory result has been satisfactory or not. From the perspective of quality management and audits, these results are indicative of the laboratories’ good performance. The results obtained in this study reflect this assertion, demonstrating that Spanish Nuclear Power Plants have achieved high percentages of satisfactory results over the nine evaluated years. These results ensure the quality of measurements from the perspective of radiological protection for the Spanish regulatory authority. However, laboratories must monitor whether the obtained results reveal any hidden signals in this satisfactory evaluation to implement corrective measures within their quality system. The PomPlots, along with the study of the RBs, can serve as a suitable tool for this purpose, and this work has shown that the information they provide collectively allows for a proper analysis of result trends. Therefore, the findings of this study are very satisfactory from the organizer’s evaluation standpoint, ensuring laboratory quality, and have shown that only a small percentage of them were statistically different from the reference values.

This work has shown that, although on numerous occasions the conclusions that can be reached through a study with a sample of the data with respect to the population of results may be equivalent, it is necessary for researchers to be aware of whether we are investigating a number of values considered as samples or as populations. Both statistical significance and conclusions may differ, and the statistical scope of our findings must be clearly stated.

## Supplementary Information

Below is the link to the electronic supplementary material.

ESM 1 (DOCX 1.02 MB)

## Data Availability

No datasets were generated or analysed during the current study.
