# Supplementary material for: Temporal evolution of the performance evaluation of the laboratories at Spanish nuclear power plants in water samples
Source: Environ Monit Assess. 2025 Mar 21;197(4):449. doi: 10.1007/s10661-025-13851-8 (PMC11928374; doi:10.1007/s10661-025-13851-8)
Supplement: Supplementary file 1 — (DOCX 1.02 MB) [file 10661_2025_13851_MOESM1_ESM.docx]

Supporting Information

Temporal evolution of the performance evaluation of the laboratories at Spanish nuclear power plants in water samples

J.A. Suárez-Navarrro^1,*^, V.Peyres^1^, A.I. Sánchez-Cabezudo^1^, N. Navarro^1^, V.M. Expósito-Suárez^1^, J. Español^2^, J.M. Arteaga^3^, M. Brun^4^, R. Miret^5^, A. Llorente^6^, M. Ibañez^7^, J.F. Benavente^1^

**Annex A1**. *Box-and-whisker plots showing the relative bias (RB) distributions for each sample of the specified radionuclide.*


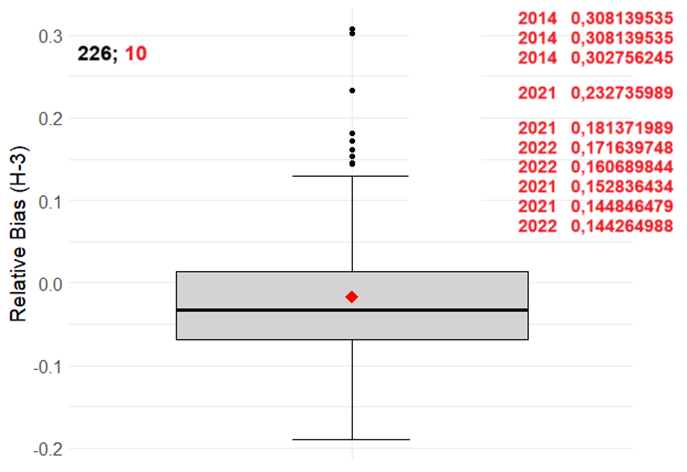


Figure A1.1 Distribution of Relative Bias (RB) for H^3^ in Sample A.


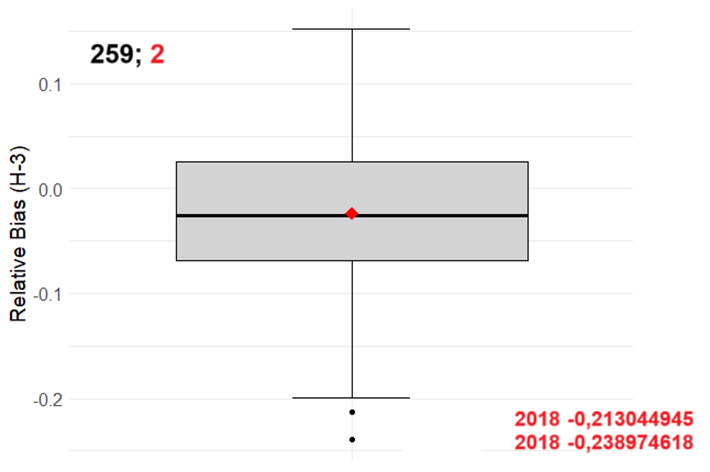


Figure A1.2 Distribution of Relative Bias for ^3^H (RB) in Sample B.


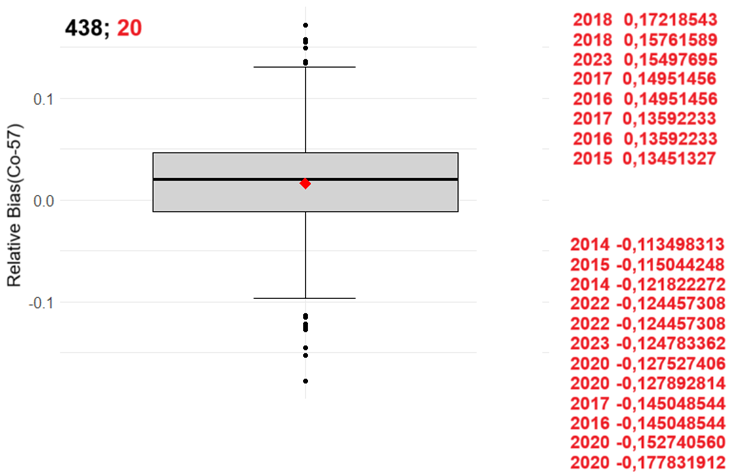


Figure A1.3 Distribution of Relative Bias (RB) for ^57^Co in Sample A.


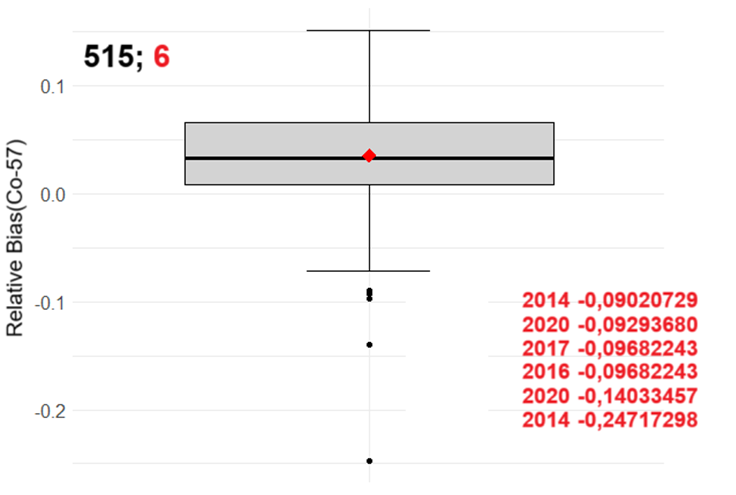


Figure A1.4 Distribution of Relative Bias (RB) for ^57^Co in Sample B.


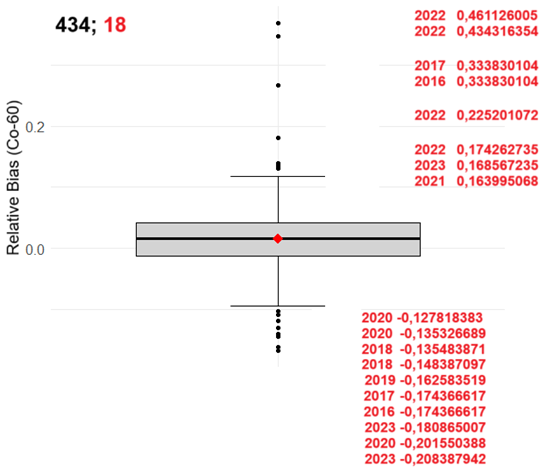


Figure A1.5 Distribution of Relative Bias (RB) for ^60^Co (1173 keV) in Sample A.


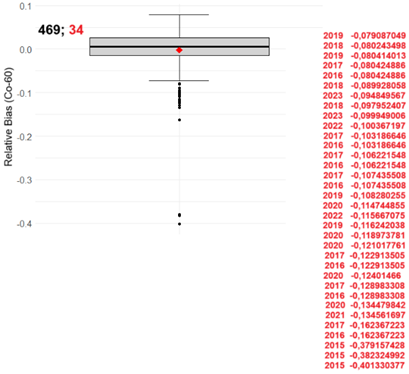


Figure A1.6 Distribution of Relative Bias (RB) for ^60^Co (1173 keV) in Sample B.


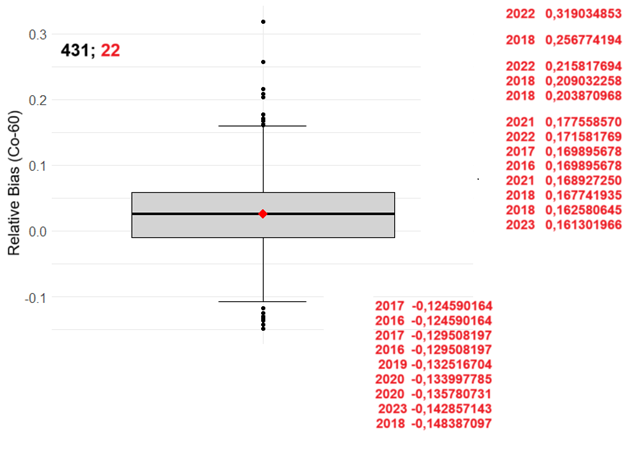


Figure A1.7 Distribution of Relative Bias (RB) for ^60^Co (1333 keV) in Sample A.


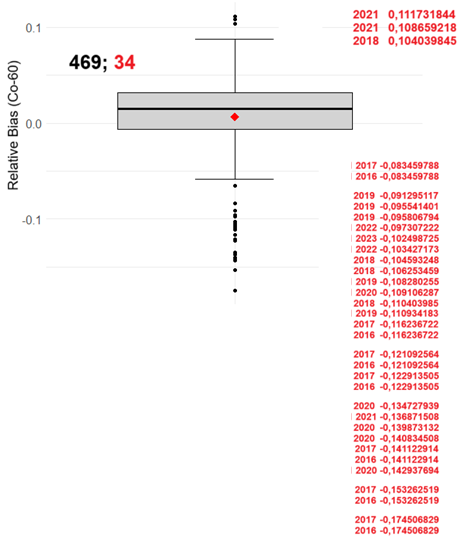


Figure A1.8 Distribution of Relative Bias (RB) for ^60^Co (1333 keV) in Sample B.


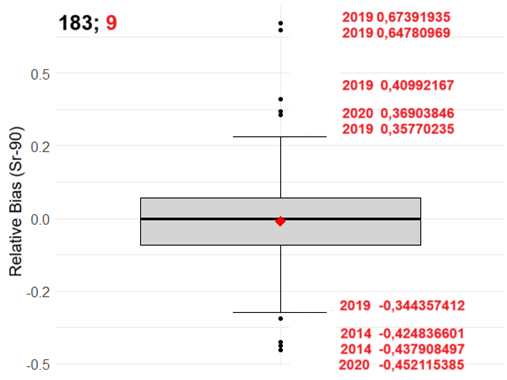


Figure A1.9 Distribution of Relative Bias (RB) for ^90^Sr in Sample A.


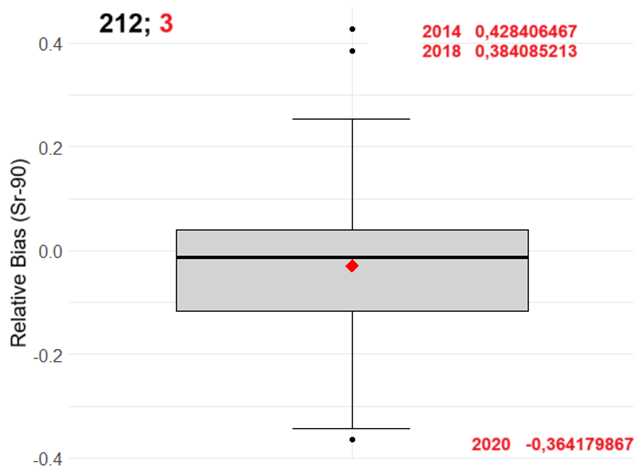


Figure A1.10 Distribution of Relative Bias (RB) for ^90^Sr in Sample B.


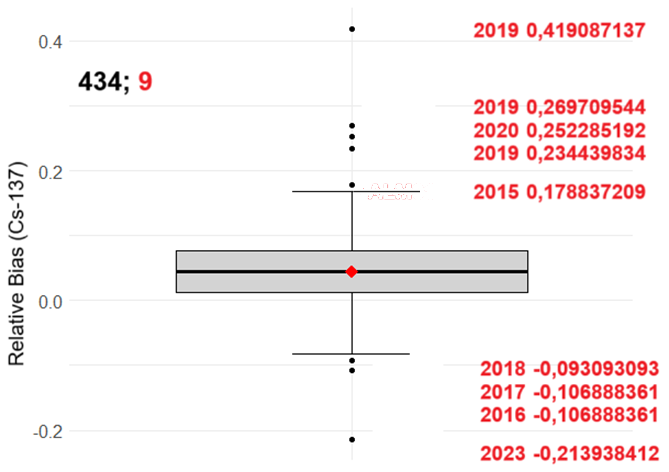


Figure A1.11 Distribution of Relative Bias (RB) for ^137^Cs in Sample A.


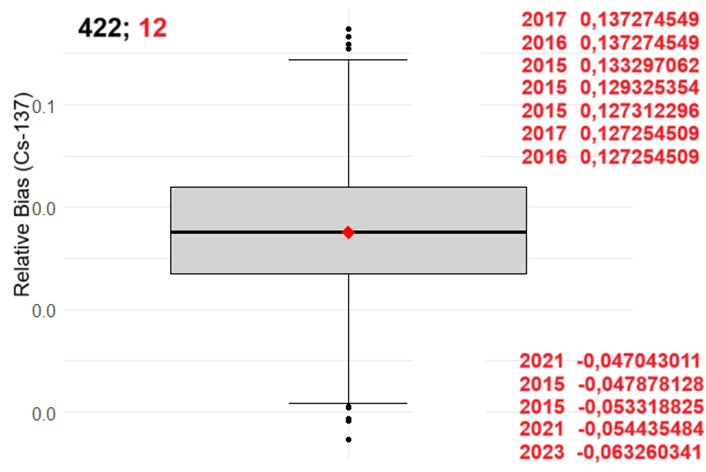


Figure A1.12 Distribution of Relative Bias (RB) for ^137^Cs in Sample B.


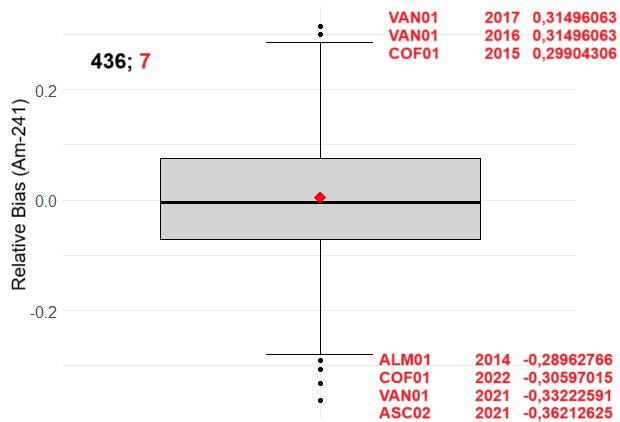


Figure A1.13 Distribution of Relative Bias (RB) for ^241^Am in Sample A.


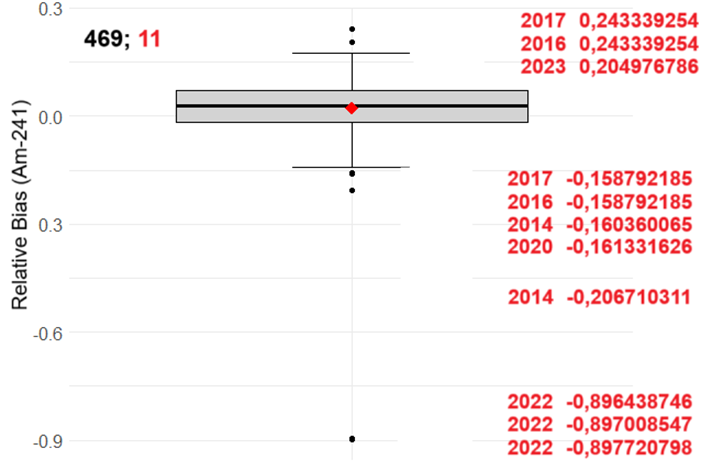


Figure A1.14 Distribution of Relative Bias (RB) for ^241^Am in Sample B.


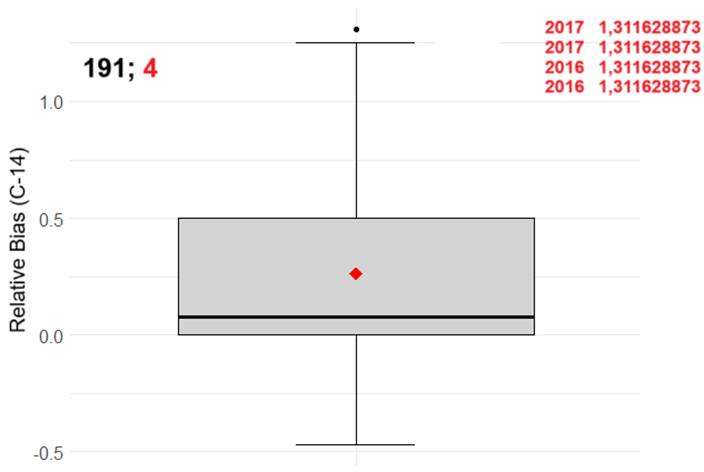


Figure A1.15 Distribution of Relative Bias (RB) for ^14^C in Sample C.
